# Quality of life among injectable and oral disease-modifying therapy users in the Pacific Northwest Multiple Sclerosis Registry

**DOI:** 10.1186/s12883-020-02016-4

**Published:** 2020-12-04

**Authors:** Tamela Stuchiner, Lindsay Lucas, Elizabeth Baraban, Kateri J. Spinelli, Chiayi Chen, Alden Smith, Lobat Hashemi, Stanley Cohan

**Affiliations:** 1 Providence Brain and Spine Institute, and Providence Multiple Sclerosis Center, Providence Health & Services, 9135 SW Barnes Rd. Suite 363, Portland, OR 97225 USA; 2 Regional Research Department, Providence Health & Services, 4805 NE Glisan St, Suite 5F40, Portland, OR 97213 USA; 3 Sanofi, 500 Kendall Street, Cambridge, MA 02142 USA

**Keywords:** Multiple sclerosis, Disease-modifying therapies, Oral DMT, Injectable DMT, Quality of life, Disability, MSIS-29, PDDS

## Abstract

**Background:**

Nine oral disease-modifying therapies (DMTs) have been approved for the treatment of multiple sclerosis (MS) in the United States. Few studies have examined self-reported quality of life (QoL) and functional status outcomes among patients who switch to oral medications from injectable MS therapies. This study compares self-reported QoL and disability status between participants switching from injectable to oral DMTs, to those who stay on injectable DMTs continuously for the same time period.

**Methods:**

Longitudinal data were assessed from relapsing MS participants in the Pacific Northwest MS Registry completing a minimum of two surveys between 2012 and 2018 with a maximum of 36 months between surveys. Stayers were defined as those who remained on injectable DMTs continuously from Time 1 to Time 2; switchers were those who switched from injectable to either fingolimod, teriflunomide or dimethyl fumarate during the same time interval. Outcomes of interest were physical and psychological QoL, measured by the Multiple Sclerosis Impact Scale (MSIS-29), and disability, measured by the Patient Determined Disease Steps (PDDS). To analyze the effect of switching to oral DMT on outcomes at Time 2, a one-to-two propensity score matching (PSM) was used to match switchers to stayers. Outcomes at Time 2 were analyzed using paired t-test for QoL scores, and Stuart Maxwell test for PDDS as a categorical variable.

**Results:**

Among 2385 participants who returned consecutive yearly surveys, 413 met the inclusion criteria for stayers and 66 for switchers. After one-to-two PSM, 124 stayers were matched to 62 switchers. Paired t-test showed no differences between switchers and stayers for physical (mean difference: − 0.41; [95% confidence interval CI: − 3.3-2.4]; *p =* 0.78) or psychological (mean difference: − 0.23; [95% CI, − 1.6- 1.1]; *p =* 0.74) QoL. Additionally, no differences were seen between switchers and stayers in self-reported disability status.

**Conclusions:**

MS registry participants who switched to an oral DMT from injectable showed no significant differences in QoL or self-reported disability status compared to those remaining on injectable DMT continuously in the same time period.

**Supplementary Information:**

The online version contains supplementary material available at 10.1186/s12883-020-02016-4.

## Background

At the time of this study, three oral disease-modifying therapies (DMTs) had received regulatory approval in the US for the treatment of multiple sclerosis (MS): fingolimod, teriflunomide, and dimethyl fumarate. Six additional oral medications, diroximel fumarate, monomethyl fumarate, siponimod, cladribine, and ozanimod, as well as a generic for dimethyl fumarate have been approved in the United States (U.S.) within the last 18 months, but were not being used by any patients in our study population. Prior to the approval of oral DMTs, injectable therapies, including interferon β-1a, interferon β-1b, and glatiramer acetate, were considered first-line medications for MS. Some patients on injectable therapies may prefer switching to oral DMTs when disease activity is not controlled, or when side effects or decline in quality of life (QoL) prompt a desire for change. In addition to therapeutic efficacy, oral DMTs offer significant benefits related to ease-of-use and potentially increased adherence, despite the requirement for daily dosing [1–3].

For many people with MS, QoL declines as the disease progresses and can be a significant burden [4]. Previous studies have found that physical and psychological QoL are influenced by progression of disease, level of disability, socio-economic factors, lifestyle choices, and DMT usage [4–6]. Few studies have examined patient reported QoL outcomes in those who have switched to oral medications from injectable therapies; existing studies assessing QoL among users of various DMTs have showed varying results.

Due to inconsistent findings and lack of published longitudinal studies of large community cohorts, we analyzed self-reported QoL and disability status from a longitudinal database collected from a regional community cohort of people with MS to help assess the impact of switching to oral from injectable DMT on QoL. The Pacific Northwest Multiple Sclerosis Registry (PNWMSR) was developed in 2007 with the objective of collecting self-reported demographic and disease-specific information from people with MS in the Pacific Northwest region of the U.S. An annual survey queries participants about access to and compliance with MS treatment options, treatment history, level of disability, comorbidities, lifestyle, and QoL. The registry is publicized through media, National MS Society events, web listings, neurology specialty clinics, and assistance from the National MS Society’s Oregon Chapter, and pharmaceutical companies. All data are self-reported through confidential surveys by participants who are age 18 or older, with a diagnosis of MS. As of December 2018, 4970 individuals with MS have registered, and 3100 are active in the registry. Because the participants’ personal data collected by the PNWMSR are confidential, and there is a minimal time commitment on the part of the participants, return rates of the follow-up survey are high, allowing us to examine self-reported aspects of this MS population longitudinally over many years. In this study, we used data from the PNWMSR to compare self-reported QoL and disability status of participants who switched from injectable to oral DMT, to those who stayed on injectable DMT continuously during the same time period.

## Methods

### Participants

Participants included in the study were registered in the PNWMSR and completed a minimum of two annual surveys between 2012 and 2018. Questions included income, education, duration and clinical pattern of MS, insurance, access to care, use of DMTs, medication adherence, current symptoms, health risk behaviors, the Patient Determined Disease Steps (PDDS) measuring disability, and the Multiple Sclerosis Impact Scale (MSIS-29), which assesses physical and psychological impact of MS on QoL [7–9].

Among registered participants with relapsing MS, two cohorts were identified: “switchers,” those who switched from injectable DMT to oral DMT, and “stayers,” those who stayed on injectable DMT. Stayers reported use of an injectable DMT for a minimum of 12 months at Time 1 and were on injectable DMT continuously through Time 2. Switchers reported use of an injectable for a minimum of 12 months at Time 1 and reported use of an oral DMT for a minimum of six months at Time 2. A minimum of > 6 months and a maximum of 36 months between Time 1 and Time 2 surveys was required for inclusion.

Participants who changed to infusion medication, stopped medication use, or did not report medication use between Time 1 and Time 2 were excluded. This study was approved by the Providence Health & Services Institutional Review Board.

### Propensity score matching

To analyze the effect of switching to oral DMT on outcomes at Time 2, we used propensity score matching (PSM) to match switchers with stayers who had a similar length of time between Time 1 and 2 surveys and who also had similar demographic and clinical characteristics at Time 1. The propensity scores were calculated using a generalized linear model with a logit link. The following matching variables were selected a priori due to their effect on outcomes in previous studies: age, sex, psychological MSIS, physical MSIS, PDDS, and health insurance availability at Time 1 and length of time between Time 1 and 2 [10–12]. If a stayer reported to be on an injectable for several surveys, the Time 1 and Time 2 surveys could be any two of their surveys as long as they met the inclusion criteria. For matching, PDDS at Time 1 was categorized as no or mild disability (0–1) or moderate to severe [2–8]. The moderate [2–4] and moderate to severe [5–8] groups were combined due to the smaller number of participants in these groups. One switcher was matched to two stayers without replacement based on closest propensity score using a caliper of 0.50 standard deviations. Only switchers and stayers with non-missing matching variables and outcomes were eligible for matching.

To determine balance between matched groups, differences in each matching covariate after PSM were calculated. Standardized mean differences of < 10% for continuous variables and absolute mean differences of < 10% for binary variables were used to determine whether the matched groups were balanced. Before PSM, continuous variables were compared between cohorts using two sample t or Wilcoxon rank sum tests, as appropriate based on normality, and categorical variables were compared with chi-square tests. After PSM, comparisons were made using paired t-tests for continuous variables, McNemar’s tests for categorical variables with two categories, and Stuart-Maxwell test for categorical variables with more than two categories.

### Statistical analysis

Psychological MSIS and physical MSIS scores at Time 2 were compared between matched switchers and stayers using paired t-tests, and PDDS at Time 2 was compared using the Stuart-Maxwell test. All tests were two-tailed with alpha equal to 0.05; *p*-values < 0.05 were considered statistically significant. R 3.3.2 software (R Foundation for Statistical Computing, Vienna, Austria) was used for statistical analyses and graphics.

### Outcomes

Three outcomes collected at Time 2 were compared between matched switchers and stayers: physical MSIS, psychological MSIS, and PDDS. The MSIS contains 29 items, 20 related to physical impact of MS on QoL and 9 related to psychological impact. Each item represents one indicator of the impact of MS on QoL (e.g. ability to do physically demanding tasks, bothered by spasms or stiffness, feeling mentally fatigued, etc.). The participant chooses a number between 1 and 4 based on the impact their disease has on that QoL item (1 = low impact, 4 = high impact). Adding each participant’s score for each test item results in a range of 20 to 80 for physical MSIS scores and 9 to 36 for psychological MSIS scores, with higher scores indicating a greater impact of MS on QoL. As recommended by MSIS scoring guidelines, participants missing greater than 50% of items on either scale were excluded from the analysis and for those missing less than 50% of items on either scale, a respondent specific mean score was imputed from the items completed [7]. The PDDS is an ordinal questionnaire that allows the participant to self-classify their level of disability on a scale of 0 to 8, with 8 being the most disabled. For this analysis, PDDS at Time 2 was categorized as none or mild disability (0–1), moderate disability [2–4], or moderate to severe disability [5–8].

### Sensitivity analysis

The PSM did not include disease duration as a matching variable because 13% (*n* = 8) of switchers were missing disease duration. However, disease duration could have an effect on outcomes at Time 2. Therefore, we performed a sensitivity analysis where disease duration was added as a matching variable.

## Results

**Fig. 1:**
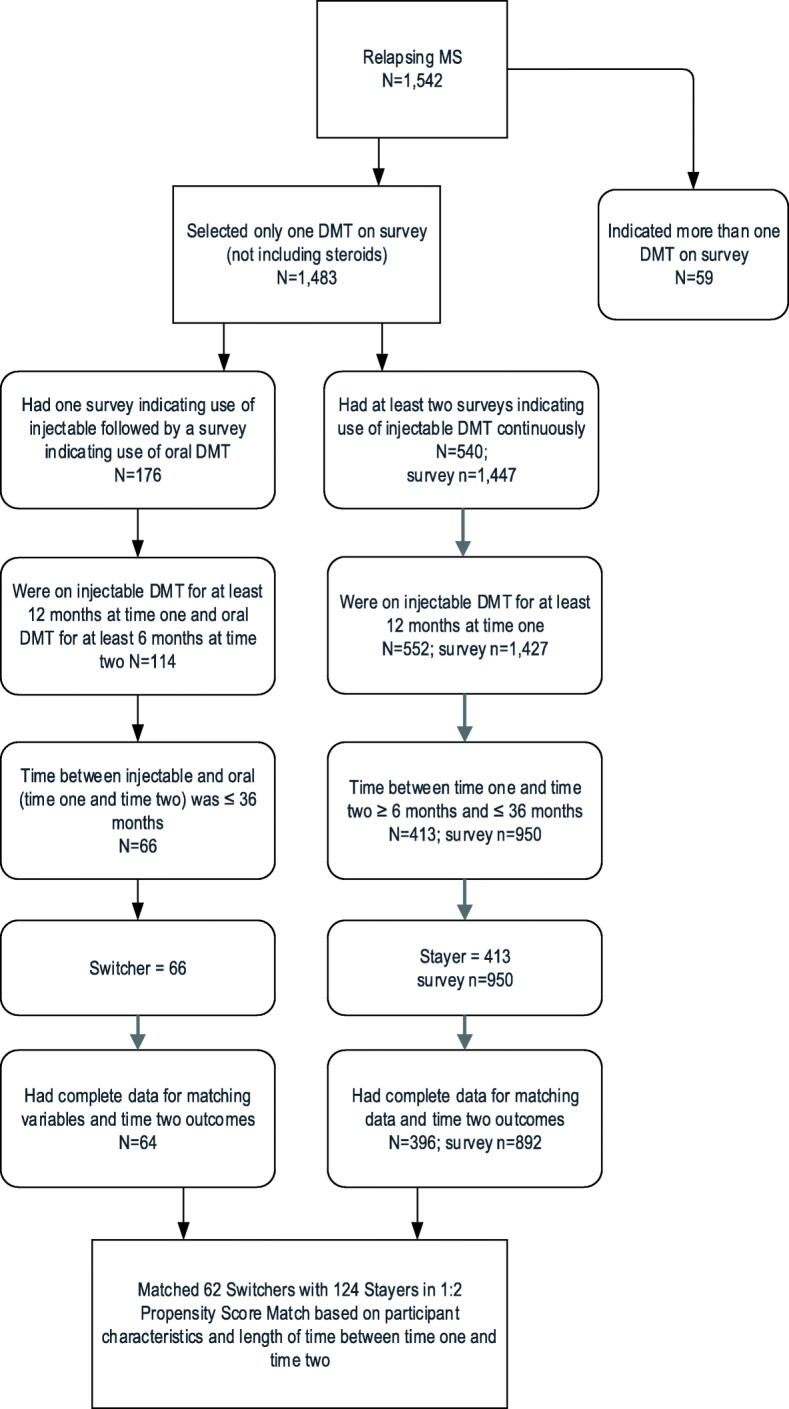
Participant and analysis flowchart

Among 2385 PNWMSR participants who completed at least two surveys between 2012 and 2018, 1542 participants had relapsing MS. Of these, 413 met the inclusion criteria for a stayer and 66 met the inclusion criteria for a switcher. Two switchers and 17 stayers had missing matching variables or Time 2 outcomes, leaving 396 stayers and 64 switchers eligible for matching (Fig. 1).

Participant characteristics at Time 1 prior to matching are reported in Table 1. There were no differences in age, disease duration, or sex. There was a numerically higher mean psychological MSIS score at Time 1 in Switchers (17.75 [±5.72] vs 16.28 [±5.96], *p* = 0.067). A significantly high number of participants in the Stayer group were of white race (*p* = 0.002) and have public health insurance (*p* = 0.01).

**Table 1:** Participant Characteristics before Matching

		Stayer	Switcher	***P*** value
		396	64	
**Mean age (SD)**		54.99 (10.34)	52.92 (12.16)	0.149
**Sex % (n)**	Female	86.6 (343)	89.1 (57)	0.734
	Male	13.4 (53)	10.9 (7)	
**Race % (n)**	White	93.7 (371)	87.5 (56)	0.002
	Non-white	3.0 (12)	0.0 (0)	
	Unknown	3.3 (13)	12.5 (8)	
**Median disease duration [IQR]**		12 [8, 18]	12 [7, 19]	0.839
**PDDS % (n)**	None or mild	41.9 (166)	51.6 (33)	0.191
	Moderate to severe	58.1 (230)	48.4 (31)	
**Work status % (n)**	Employed	46.3 (179)	55.6 (35)	0.217
	Not Employed	53.7 (208)	44.4 (28)	
**Insurance % (n)**	Public	27.0 (107)	20.3 (13)	0.01
	Private	44.2 (175)	64.1 (41)	
	None/Other/Not Reported	28.8 (114)	15.6 (10)	
**Mean MSIS Physical Score at Time 1 (SD)**		33.85 (12.49)	31.69 (9.98)	0.19
**Mean MSIS Psychological Score at Time 1 (SD)**		16.28 (5.96)	17.75 (5.72)	0.067
**Median time on drug in months at Time 1 [IQR]**		105.50 [56.50, 144.00]	95.00 [61.50, 129.75]	0.513

[*SD* standard deviation, *IQR* Interquartile range, *PDDS* Patient Determined Disease Steps, *MSIS* Multiple Sclerosis Impact Scale]

In the one-to-two PSM, 124 stayers were matched with 62 switchers. Standardized mean differences were < 10% for continuous variables and absolute mean differences were < 10% for binary variables (Supplemental Figure 1). After matching, there were no statistically significant differences in matching variables between participants, however white race was higher in Stayers and disease duration was higher in Switchers (*p* = 0.017 and *p* = 0.038 respectively) (Table 2).

**Table 2:** Participant Characteristics after Matching

		Stayer	Switcher	***P*** value
		124	64	
**Mean age (SD)**		52.56 (10.51)	53.18 (11.96)	0.617
**Sex % (n)**	Female	87.9 (109)	88.7 (110)	1.000
	Male	12.1 (15)	11.3 (14)	
**Race % (n)**	White	92.7 (115)	90.3 (112)	0.017
	Non-white	4.0 (5)	0.0 (0)	
	Unknown	3.2 (4)	9.7 (12)	
**Median disease duration [IQR]**		11 [7, 15]	12 [7, 19]	0.038
**PDDS % (n)**	None or mild	44.4 (55)	51.6 (64)	0.306
	Moderate to severe	55.6 (69)	48.4 (60)	
**Work status % (n)**	Employed	52.9 (63)	55.7 (68)	0.435
	Not Employed	47.1 (56)	44.3 (54)	
**Insurance % (n)**	Public	31.5 (39)	21.0 (26)	0.114
	Private	57.3 (71)	62.9 (78)	
	None/Other/Not Reported	11.3 (14)	16.1 (20)	
**Mean MSIS Physical Score at Time 1 (SD)**		32.12 (11.67)	31.76 (10.09)	0.779
**Mean MSIS Psychological Score at Time 1 (SD)**		17.61 (6.62)	17.55 (5.66)	0.916
**Median time on drug in months at Time 1 [IQR]**		108.00 [50.50, 139.50]	97.50 [ 65.00, 132.00]	0.955

[*SD* standard deviation, *IQR* Interquartile range, *PDDS* Patient Determined Disease Steps, *MSIS* Multiple Sclerosis Impact Scale]

**Fig. 2:**
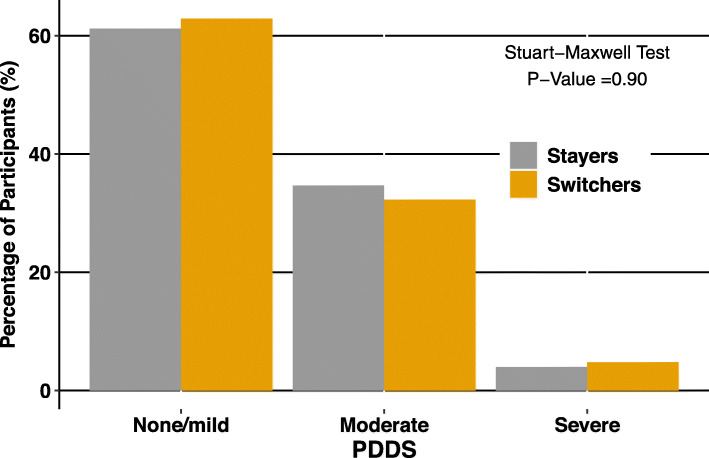
Time 2 Disability Outcomes After Propensity Score Matching. PDDS disability scores were grouped into mild disability (0–1), moderate disability (2–4) or moderate to severe disability (5–8). Stayers and switchers were compared in each group

Physical and psychological QoL scores for matched participants at Time 2 are reported in Table 3. After matching, there were no significant differences between stayers and switchers for impact of MS on physical (mean difference: − 0.41; [95% confidence interval, CI: − 3.3-2.4; *p* = 0.78] or psychological (mean difference: -0.23 [95% CI: − 1.6-1.1]; *p* = 0.74) QoL. PDDS at Time 2 also showed no significant differences between switchers and stayers after matching (Fig. 2).

**Table 3:** Time 2 QoL Outcomes after Propensity Score Matching

	Stayers (***N*** = 124)	Switchers (***N*** = 64)	Mean Difference (95% CI)	Paired t-test ***P*** -value
Physical MSIS-29, mean (SD)	32.6 (±11.6)	32.2 (±10.9)	−0.41 (−3.25, 2.43)	0.78
Psychological MSIS-29, mean (SD)	16.5 (±6.6)	16.2 (±4.9)	−0.23 (−1.56, 1.11)	0.74

**Fig. 3:**
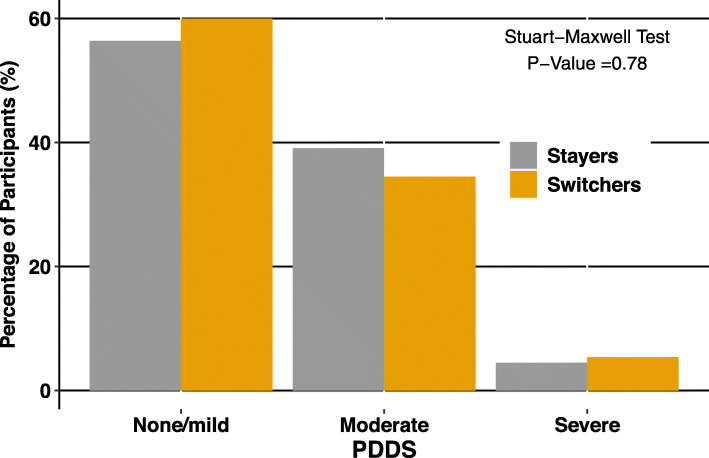
Time 2 Disability Outcomes After Propensity Score Matching with Sensitivity Analysis including Disease Duration as a Matching Variable. PDDS disability scores were grouped into mild disability (0–1), moderate disability (2–4) or moderate to severe disability (5–8). Stayers and switchers were compared in each group

Sensitivity analysis with inclusion of disease duration as a matching variable resulted in a one-to-two PSM with 110 stayers with 55 switchers. Standardized mean differences were < 11% for continuous variables and absolute mean differences were < 10% for binary variables. There were no significant differences after matching in the sensitivity analysis in physical QoL (mean difference: -0.70 [95% CI: − 3.8-2.4] *p* = 0.65), in psychological QoL (mean difference: -1.49 [95% CI: − 3.0-0.03] *p* = 0.06; or PDDS (Table 4, Fig. 3).

**Table 4:** Time 2 QoL Outcomes after Propensity Score Matching with Sensitivity Analysis

	Stayers (***N*** = 110)	Switchers (***N*** = 55)	Mean Difference (95% CI)	Paired t-test ***P*** -value
Physical MSIS-29, mean (SD)	33.5 (±10.7)	32.8 (±11.4)	− 0.70 (− 3.77, 2.36)	0.65
Psychological MSIS-29, mean (SD)	17.7 (±6.4)	16.3 (±5.0)	−1.49 (−3.01, 0.03)	0.06

## Discussion

Participants in the PNWMSR who stayed on injectable DMTs reported no significant differences in physical or psychological MSIS when compared to those who switched to an oral DMT at Time 2 after propensity score matching. The numeric difference in the psychological QoL at Time 2 suggests a better outcome for switchers than stayers, although the difference did not achieve statistical significance. Given the wide confidence intervals, this failure to reach statistical significance (*p* = 0.06) may reflect the relatively small size of the stayer cohort, a potential shortcoming that may be addressed in future studies.

Additionally, no differences were seen in reported disability status between those who stayed on injectable versus those who switched to an oral medication.

To our knowledge, this is the first longitudinal study to provide clinicians with data on the long-term impact of changing from an injectable to an oral medication on MS patients’ perceived QoL and disability. While some of the most notable reported differences between injectable and oral DMT have been superior adherence and persistence to oral therapy, our survey design did not allow us to assess adherence or persistence [1, 2, 13]. Despite previous data on better adherence for oral DMTs, our study found no significant differences in impact of switching to oral DMTs on patient self-reported QoL and disability status.

Self-perceived disability status and physical and psychological QoL can play a role in medication adherence and persistence. McKay et al. reported that longer disease duration, mild disability status, and perceived cognitive difficulties were all associated with non-adherence [14]. In addition, Devonshire et al. found that adherent patients had significantly better scores on QoL and less psychological issues [15]. Our findings are at odds with some previously reported studies, adding to the knowledge base that may assist both MS patients and their health care providers in the decision-making process regarding medication choices.

Comparative DMT efficacy measures were not part of our study design and were beyond the scope of this project. Future studies should directly address the relationship between medication adherence and self-reported measures of perceived efficacy, QoL, and disability status among oral and injectable DMT users. In addition, future studies should include the recently U.S.-approved oral medications cladribine, siponimod, and diroximel fumarate, as their use becomes more widespread and of prolonged duration.

Prior studies examining differences in QoL among users of injectable versus oral DMT, as well as those who switch from injectable to oral DMT, have had conflicting results [16–20]. The Evaluate Patient Outcomes trial and related post hoc analyses found that patients switching from injectable to the oral DMT fingolimod had improved patient-reported and physician-reported outcomes at six months, including global satisfaction with treatment, improved QoL, and decreased fatigue and depression [16, 18, 19]. A study examining patient-reported outcomes in relapsing MS patients switching to teriflunomide from other DMTs found patients had sustained stable QoL, using the Multiple Sclerosis International Quality of Life (MusiQoL) [17]. In contrast, a study comparing QoL among relapsing MS patients by use of DMT, utilizing the Functional Assessment of Multiple Sclerosis (FAMS) and the Expanded Disability Status Scale (EDSS), found that there were no significant differences between users of interferon β-1b, fingolimod, and natalizumab [20].

Our results contribute to the literature in several ways. First, our study was conducted using longitudinal data from a community-based cohort, utilizing self-reported QoL and disability. Second, although limited to participants from the Pacific Northwest, our sample was more representative of the general MS population, and not subject to the selection bias inherent in controlled clinical trials or demographic bias when patients are recruited from specific clinics or MS Centers, in that participation was voluntary and participants were recruited via multiple techniques and sources, including local chapters of the National MS Society. This allows for a more broadly representative sample. When comparing recently published average MSIS-29 values from another registry with the ones from our registry, MSIS-29 scores (physical) are higher in our study, while, conversely, MSIS-29 psychological scores are lower in our study, highlighting variability among reported MSIS data in published studies [21]. One potential contributor to the seemingly low MSIS scores is that the population was comprised of patients with the relapsing form of disease, who were receiving disease modifying therapy, thus eliminating potentially higher scores associated with the greater disability accompanying the progressive phenotype of the disease. Indeed, the relatively lower numbers of patients with moderate [2–4] and moderate to severe [5–8] PDDS scores in this study supports this notion.

There are several limitations of this study. First, this was an observational rather than a randomized design utilizing self-reported measures. However, the nature of the design allowed our participants to be followed for up to 36 months in a real-world setting, whereas analyses published previously have been of shorter duration and thus provide no information on longer-term or sustained benefits of switching to oral DMTs, or were limited to the protocol-determined patient selection restrictions of controlled clinical trials [16, 18, 19]. Second, there may be potential shortcomings associated with incomplete or missing data. To address this limitation for the physical and psychological MSIS, participants missing greater than 50% of items on either scale were excluded from the analysis. For those missing less than 50% of items on either scale, a respondent specific mean score was imputed from the items completed [7]. Third, the participants were from a specific geographical region and thus results may not be generalizable to those living in other areas, or in which greater racial or ethnic diversity is present in the MS population. However, the design allowed for capture of a very high percentage of patients living within the registry catchment area. Fourth, reasons for switching DMT were not collected. Further studies could explore the relationship between reasons for switching and patient outcomes. Fifth, adherence data was not collected, thus impact of the known adherence-related benefit of oral DMTs could not be assessed. Lastly, three new oral agents have been approved since the last data collection cycle, and thus their impact cannot be reported in the current study but will be included in future assessments.

We believe that our study provides meaningful insights by showing a lack of impact of switching from injectable to oral DMTs on MS participants’ self-reported QoL and disability status, perhaps tempering what may be overly sanguine assumptions regarding oral DMTs in the areas with which this study is concerned. In addition, our study supports the utility of community-based sources of data for MS research.

## Conclusions

In our large, regional, community-based MS registry, participants making the transition from injectable to oral DMTs showed no significant differences in self-reported outcomes for physical and psychological QoL and disability compared to those who stayed on injectable DMT. As the duration and number of subjects using oral DMTs for MS grows, additional outcomes analyses that include differentiations between the various oral agents, inclusion of more recently approved oral DMTs, higher efficacy parenteral DMTs, adherence and discontinuation analyses, and side effect profiles will provide critically important analytic opportunities.

## Supplementary Information

**Additional file 1.**

## Data Availability

The datasets generated and/or analyzed during the current study are available in the Pacific Northwest MS Registry repository, but restrictions apply to the availability of these data, which were used under license for the current study, and so are not publicly available.

## Abbreviations

95% CI: 95% confidence intervals
DMT: disease-modifying therapy
EDSS: Expanded Disability Status Scale
FAMS: Functional Assessment of Multiple Sclerosis
MSIS-29: Multiple Sclerosis Impact Scale
PDDS: Patient Determined Disease Steps
PSM: propensity score matching
QoL: quality of life

## References

[1] Agashivala N, Wu N, Abouzaid S, Wu Y, Kim E, Boulanger L (2013). Compliance to fingolimod and other disease modifying treatments in multiple sclerosis patients, a retrospective cohort study. BMC Neurol.

[2] Bergvall N, Petrilla AA, Karkare SU, Lahoz R, Agashivala N, Pradhan A (2014). Persistence with and adherence to fingolimod compared with other disease-modifying therapies for the treatment of multiple sclerosis: a retrospective US claims database analysis. J Med Econ.

[3] Higuera L, Carlin CS, Anderson S (2016). Adherence to disease-modifying therapies for multiple sclerosis. J Manag Care Spec Pharm.

[4] Janzen W, Turpin KV, Warren SA, Marrie RA, Warren KG (2013). Change in the health-related quality of life of multiple sclerosis patients over 5 years. Int J MS Care..

[5] Brola W, Sobolewski P, Fudala M, Flaga S, Jantarski K, Ryglewicz D (2016). Self-reported quality of life in multiple sclerosis patients: preliminary results based on the polish MS registry. Patient Prefer Adherence.

[6] Jelinek GA, De Livera AM, Marck CH, Brown CR, Neate SL, Taylor KL (2016). Lifestyle, medication and socio-demographic determinants of mental and physical health-related quality of life in people with multiple sclerosis. BMC Neurol.

[7] Hobart J, Cano S (2009). Improving the evaluation of therapeutic interventions in multiple sclerosis: the role of new psychometric methods. Health Technol Assess.

[8] Hohol MJ, Orav EJ, Weiner HL (1995). Disease steps in multiple sclerosis: a simple approach to evaluate disease progression. Neurology..

[9] Hohol MJ, Orav EJ, Weiner HL (1999). Disease steps in multiple sclerosis: a longitudinal study comparing disease steps and EDSS to evaluate disease progression. Mult Scler.

[10] Braune S, Grimm S, van Hovell P, Freudensprung U, Pellegrini F, Hyde R (2018). Comparative effectiveness of delayed-release dimethyl fumarate versus interferon, glatiramer acetate, teriflunomide, or fingolimod: results from the German NeuroTransData registry. J Neurol.

[11] Ontaneda D, Nicholas J, Carraro M, Zhou J, Hou Q, Babb J (2019). Comparative effectiveness of dimethyl fumarate versus fingolimod and teriflunomide among MS patients switching from first-generation platform therapies in the US. Mult Scler Relat Disord..

[12] Prosperini L, Lucchini M, Haggiag S, Bellantonio P, Bianco A, Buscarinu MC (2018). Fingolimod vs dimethyl fumarate in multiple sclerosis: a real-world propensity score-matched study. Neurology..

[13] Thach AV, Brown CM, Herrera V, Sasane R, Barner JC, Ford KC (2018). Associations between treatment satisfaction, medication beliefs, and adherence to disease-modifying therapies in patients with multiple sclerosis. Int J MS Care.

[14] McKay KA, Tremlett H, Patten SB, Fisk JD, Evans C, Fiest K (2017). Determinants of non-adherence to disease-modifying therapies in multiple sclerosis: a cross-Canada prospective study. Mult Scler.

[15] Devonshire V, Lapierre Y, Macdonell R, Ramo-Tello C, Patti F, Fontoura P (2011). The global adherence project (GAP): a multicenter observational study on adherence to disease-modifying therapies in patients with relapsing-remitting multiple sclerosis. Eur J Neurol.

[16] Calkwood J, Cree B, Crayton H, Kantor D, Steingo B, Barbato L (2014). Impact of a switch to fingolimod versus staying on glatiramer acetate or beta interferons on patient- and physician-reported outcomes in relapsing multiple sclerosis: post hoc analyses of the EPOC trial. BMC Neurol.

[17] Coyle PK, Khatri B, Edwards KR, Meca-Lallana JE, Cavalier S, Rufi P (2018). Patient-reported outcomes in patients with relapsing forms of MS switching to teriflunomide from other disease-modifying therapies: results from the global phase 4 Teri-PRO study in routine clinical practice. Mult Scler Relat Disord..

[18] Fox E, Edwards K, Burch G, Wynn DR, LaGanke C, Crayton H (2014). Outcomes of switching directly to oral fingolimod from injectable therapies: results of the randomized, open-label, multicenter, evaluate patient OutComes (EPOC) study in relapsing multiple sclerosis. Mult Scler Relat Disord.

[19] Hunter SF, Agius M, Miller DM, Cutter G, Barbato L, McCague K (2016). Impact of a switch to fingolimod on depressive symptoms in patients with relapsing multiple sclerosis: an analysis from the EPOC (evaluate patient OutComes) trial. J Neurol Sci.

[20] Yahya RN, Kasim AA, Al Gawwam AA (2018). Comparing the quality of life among patients with relapsing remitting multiple sclerosis in Iraq using different disease modifying therapies. Iraqi J Pharm Sci (IJPS).

[21] McKay KA, Ernstsson O, Manouchehrinia A, Olsson T, Hillert J (2020). Determinants of quality of life in pediatric- and adult-onset multiple sclerosis. Neurology..

