# Supplementary figures and images for: Quality of life among injectable and oral disease-modifying therapy users in the Pacific Northwest Multiple Sclerosis Registry
Source: BMC Neurol. 2020 Dec 4;20:439. doi: 10.1186/s12883-020-02016-4 (PMC7716591; doi:10.1186/s12883-020-02016-4)

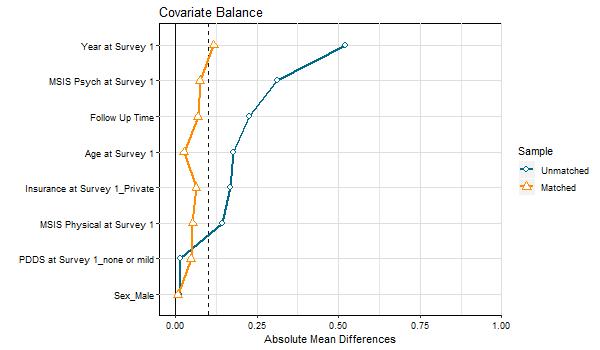

Supplement: Supplementary file 1 — Additional file 1. [file 12883_2020_2016_MOESM1_ESM.jpg]
